# Differences in Histological Subtypes of Invasive Lobular Breast Carcinoma According to Immunohistochemical Molecular Classification

**DOI:** 10.3390/diagnostics14060660

**Published:** 2024-03-21

**Authors:** Ivan Ilić, Jana Cvetković, Ratko Ilić, Ljubiša Cvetković, Aleksandar Milićević, Stefan Todorović, Pavle Ranđelović

**Affiliations:** 1 Center for Pathology and Pathological Anatomy, University Clinical Center Niš, Faculty of Medicine, University of Niš, 18000 Niš, Serbia; ilic.ratko@gmail.com (R.I.); ackom88@gmail.com (A.M.); 2 Department for Pathology, General Hospital Leskovac, 16000 Leskovac, Serbia; jana.cvetkovic92@gmail.com (J.C.); ljubisapatolog@gmail.com (L.C.); 3 Clinic of Neurology, University Clinical Center Niš, 18000 Niš, Serbia; todorovicstefan815@gmail.com; 4 Department of Physiology, Faculty of Medicine, University of Niš, 18000 Niš, Serbia; pavleus@gmail.com

**Keywords:** invasive lobular carcinoma, estrogen and progesterone receptors, immunohistochemistry, molecular subtypes, triple-negative breast cancer

## Abstract

The technical complexity of gene expression profiling in routine practice has necessitated the use of surrogate molecular classification of breast cancer, based on immunohistochemical analyses. Background and objectives: The aim of this study was to compare the differences between histological and molecular subtypes of invasive lobular carcinoma (ILC) of the breast, in order to be able to predict the behavior and prognosis of the disease, as well as to effectively determine therapy. Material and Methods: This study included 263 cases of breast ILC diagnosed over a seven-year period. The diagnosis of invasive lobular carcinoma is based on the characteristic growth pattern and phenotype of cancer cells with the respective subtypes: classic, alveolar, solid, tubulolobular, pleomorphic and mixed lobular type. The examined cases were divided into five groups according to molecular classification based on the expression of ER, PR, HER2 and Ki67 immunohistochemical markers. Results: It was found that the pleomorphic subtype occurred statistically significantly less often as the luminal A subtype compared to others (*p* = 0.00027), and the HER2-enriched subtype occurred statistically significantly more often in the pT4 stage (*p* = 0.024). Conclusions: The results of this study significantly singled out the luminal A subtype, and among them classic ILC, as the subtype with the most favorable expression ratio of the investigated predictive/prognostic immunohistochemical markers.

## 1. Introduction

Invasive lobular carcinoma (ILC) is an invasive breast carcinoma (IBC) that consists of discohesive cells, which are usually individually arranged or have a linear growth pattern. ILCs often present as irregular and ill-defined tumors, which are sometimes difficult to define macroscopically due to diffuse infiltrative growth [1]. The size of ILCs is also difficult to determine, although some studies have reported them to be slightly larger than those of IBC-NSTs [1,2].

The diagnosis of invasive lobular carcinoma is based on the characteristic growth pattern and phenotype of cancer cells with the respective subtypes: classic, alveolar, solid, tubulolobular, pleomorphic and mixed lobular type [1,2,3].

In general, up to 15% of palpable breast cancers are not registered on mammography, but most can be identified with targeted ultrasound. On the other hand, MRI is not the most specific, but it is the most sensitive method in the detection of breast cancer and some centers routinely use it after the initial diagnosis of lobular cancer for breast cancer staging purposes. Also, it is more useful in the diagnosis of multifocal and multicentric ILC, but it can unfortunately lead to false-positive results due to tumor size overestimation [4,5,6].

The treatment of breast cancer is primarily based on clinical and TNM staging, which depends on the size of the primary tumor, the presence of regional lymph node metastases and their number, as well as the presence of distant organ metastases. Differences in prognosis may exist between two tumors of the same clinical stage and the same pathohistological characteristics. That is why priority is given to individualized therapy, which depends on the molecular characteristics of breast cancer, which has been confirmed by many studies on breast cancer genotyping, emphasizing that different molecular subtypes have varying prognoses. Standard prognostic factors depend on patient age, disease stage, tumor grade, histological type, status of resection margins and presence of lymphovascular invasion [1]. Additional well-known prognostic markers, and at the same time predictors of therapeutic response, are ER and HER2 receptors [7,8,9], which is why determining the status of ER, PR and HER2 hormone receptors has been deemed as a necessary factor for making further decisions about breast cancer therapy [1].

Breast cancer is heterogeneous at the molecular level, with different patterns of gene expression leading to differences in behavior and prognosis [10,11]. Over the past few years, considerable efforts have been made to describe and classify breast cancer at the molecular level, in order to effectively tailor treatment. However, due to time and cost constraints in the vast majority of healthcare systems, surrogate molecular classification of breast cancer is still largely based on the immunohistochemical assessment of biomarkers (ER, PR, HER2 and Ki-67) [1,12].

The expression of ER and PR receptors is present in about 75% of all breast cancers and they are definite indicators of response to antihormonal therapy. The threshold value of ER and PR receptor expression was estimated at a minimum of 1% of immunohistochemically positive nuclei. Most ER-positive breast cancers also show PR co-expression, while a small percentage of breast cancers show positivity for individual hormone receptors (ER or PR). It seems that the tumors with individual hormone receptor positivity are more aggressive and less responsive to antihormonal therapy compared to both ER- and PR-positive breast cancers. Every seventh breast cancer overexpresses HER2 receptors with amplification of the corresponding gene, which can be tested by a combination of immunohistochemical and in situ DNA hybridization techniques [1,13,14,15]. The technical complexity of gene expression profiling in routine practice has conditioned the generally accepted use of surrogate molecular classification of breast cancer, which is based on immunohistochemical analyses [15,16]. Immunohistochemical analysis of ER, PR, HER2 and Ki-67 is used to identify the following subtypes: (1) luminal A-like; (2) luminal B-like, HER2 negative; (3) luminal B-like, HER2 positive; (4) HER2 positive, non-luminal; (5) triple negative [16,17].

Luminal A and luminal B subtypes are characterized by a molecular profile of gene expression that mostly resembles normal cells of the luminal layer of the mammary duct, as well as by other genes associated with ER activation. Luminal A subtype is the most common molecular subtype, which expresses ER/PR, does not overexpress HER2 and its Ki-67 proliferative index is low. This subtype accounts for almost half of all invasive breast cancers [18], is typically low grade and has the best prognosis of all other molecular subtypes.

The classical subtype of ILC in most cases expresses both ER and PR without excessive expression/amplification of HER2, so, according to the expression of these biomarkers and according to gene expression, in 85% of cases, they are classified into the luminal A molecular subtype [1], and certain studies with applied immunohistochemical analyses [19] reported that they can be of the luminal B subtype, and rarely of the HER2-enriched and triple-negative molecular subtypes. The pleomorphic subtype of ILC, especially the apocrine variant, often has a loss of ER/PR expression with overexpression of HER2 [20].

## 2. Materials and Methods

In a seven-year period, 2418 cases of invasive breast cancer were diagnosed at the Niš Teaching Hospital, of which 263 (10.88%) were invasive lobular breast cancer. The analyzed samples used for immunohistochemical analyses were obtained by excisional biopsies and mastectomy with axillary dissection. The samples were processed using a standard procedure and embedded in paraffin and archived with other clinical documentation in the Pathology Department of the Niš Teaching Hospital. The Ethical Committee of the Medical School of the University of Niš approved the study (No. 12-3627-2/3). The diagnosis of invasive lobular carcinoma is based on the characteristic growth pattern and phenotype of cancer cells with their described subtypes: classic, alveolar, solid, tubulolobular, pleomorphic and mixed lobular type, according to the WHO’s classification. The classic subtype of ILC is characterized by the proliferation of small, poorly cohesive cells that are individually scattered in the fibrous connective tissue or are arranged in the form of linear bands that invade the stroma, with low to moderate nuclear grade, as well as a low mitotic index. In this subtype of ILC, ER immunoreactivity is high and HER2 is negative/non-amplified. Pleomorphic lobular carcinoma (PLC) also has a characteristic growth pattern as a classical subtype of ILC but with a higher degree of pleomorphism and a higher mitotic index. The solid subtype of ILC is characterized by typical non-cohesive, small cells with a lobular morphology, which grow in fields and have a higher frequency of mitoses than the classical subtype. Cells of the alveolar subtype of ILC are generally arranged in spherical aggregates of at least 20 cells. A mixture of tubules and small uniform cells arranged in single files constitutes the tubulolobular subtype of ILC. A mixed subtype of ILC consists of a mixture of the classic subtype and one or more other subtypes of ILC [1]. All cases of lobular carcinoma included in this study had unequivocal micromorphological features of invasive lobular carcinoma, as confirmed by three pathologists, so the use of E-cadherin immunostaining was not required.

As there is no agreement about a clear definition of multifocality/multicentricity [21], the presence of two or more invasive lobular carcinomas with at least 5 cm of normal tissue between them was adopted as a multicentricity parameter, and the local presence of lobular or ductal carcinoma in situ was also part of the diagnosis of multifocal breast carcinoma. Immunohistochemical staining was performed from one paraffin block that was selected based on standard HE staining. Tissue samples were cut from the paraffin block to a thickness of 4 μm and placed on superfrost slides for immunohistochemical staining for the presence of: estrogen receptors (Monoclonal Mouse Anti-Human Estrogen Receptor a (ER); Clone 1D5; Code N1575, Ready-to-use; DAKO, Glostrup, Denmark), progesterone receptors (Monoclonal Mouse Anti-Human Progesterone Receptor (PR); Clone PgR 636; Code N1630, Ready-to-use; DAKO, Glostrup, Denmark), HER-2 receptors (Polyclonal Rabbit AntiHuman c-erbB-2 Oncoprotein; Code A0485, 1:250–1:350; DAKO, Glostrup, Denmark) and Ki-67 antigen (Monoclonal Mouse Anti-Human Ki-67 Antigen, Clone MIB-1; Code N1633, Ready-to-use; DAKO, Glostrup, Denmark). Immunohistochemical reactions were visualized using diaminobenzidine (DAB) and contrast staining with Mayer’s hematoxylin. Despite the cited recommendations [22], we decided on a more reliable determination of positivity, in order to avoid cases with borderline positivity or false-positive cases. Invasive lobular carcinomas were considered ER or PR positive if at least 1% of cancer cells had strong nuclear staining or 10% of tumor cells had weak to moderate nuclear staining. HER2 positive invasive lobular carcinomas had more than 10% of tumor cells with complete intense membranous staining. Unequivocal (HER2++) cases were reprocessed from the same tissue molds by CISH staining using the HER2 CISH pharmDx Kit (Code SK109, DAKO, Glostrup, Denmark) and further classified as non-amplified HER2 status (1–5 signal dots per tumor cell nucleus), polyploid HER2 status (6–10 signal dots per nucleus in over 50% of tumor cells or smaller clusters) or amplified HER2 status (over 10 separate signal dots per nucleus or clusters in over 50% of tumor cells), and then classified into appropriate molecular subtypes. Detailed recommendations on test performance and interpretation are available in country-specific guidelines, such as those published by ASCO/CAP [9]. Ki-67 proliferative index is not routinely evaluated in breast cancer, but its importance in determining molecular subtypes is well known [1]. The Ki-67 proliferative index was determined by counting a minimum of 200 stained nuclei of tumor cells/area in relation to the total number of tumor cells/area. For cases where Ki-67 staining was homogeneous, at least two random high-power fields were selected, and for cases with heterogeneous Ki-67 staining, the average of the high-power fields with the strongest (hot spot) and weakest (cold spot) expression was calculated. A value of 14% of positive nuclear staining of tumor cells was considered, the boundary between a high and low Ki-67 index.

**Figure 1:**
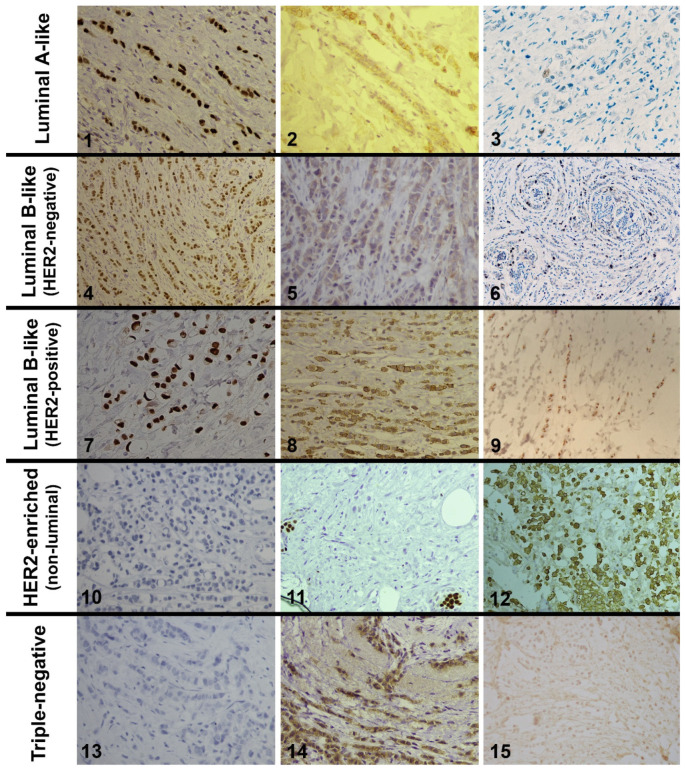
1—Strong nuclear expression of estrogen receptors in the classical ILC (LSAB × 400); 2—Weak membrane staining of HER2 oncoprotein (negative reaction) (LSAB × 400); 3—Low Ki-67 proliferative index (LSAB × 200); 4—Moderate nuclear expression of estrogen receptors in the classical ILC (LSAB × 200); 5—Weak and incomplete membrane staining of HER2 oncoprotein (negative reaction) (LSAB × 400); 6—High Ki-67 proliferative index (about 25%) (LSAB × 100); 7—Strong nuclear expression of estrogen receptors in the pleomorphic ILC (LSAB × 400); 8—Weak to moderate membranous staining in more than 10% tumor cells (unequivocal score 2+) (LSAB × 400); 9—CISH amplified HER2 status (clusters of signal points in over 50% of tumor cell nuclei) (LSAB × 400); 10—Negative expression of estrogen receptors in the pleomorphic ILC (LSAB × 400); 11—Negative expression of progesterone receptors with positive internal control of surrounding ducts (LSAB × 400); 12—Strong and complete membrane expression of HER2 oncoprotein in the pleomorphic ILC (LSAB × 200); 13—Negative expression of estrogen receptors (LSAB × 400); 14—Moderately strong aberrant cytoplasmic and incomplete membranous staining of HER2 oncoprotein in more than 10% tumor cells is considered negative (LSAB × 400); 15—CISH non-amplified HER2 status (several signal points in the tumor cell nuclei) (LSAB × 400).

The examined cases of invasive lobular carcinomas were divided into five groups according to the molecular classification of subtypes as per the above-mentioned immunohistochemical markers, and according to the recommendations of the 13th St. Gallen International Breast Cancer Conference (2013) [17] on: (1) luminal A-like subtype (ER: positive; PR: positive; HER2: negative; Ki-67 proliferation index: low); (2) luminal B-like, HER2-negative subtype (ER: positive; HER2: negative; Ki-67 proliferation index: high; PR: negative or low); (3) luminal B-like, HER2-positive subtype (ER: positive; HER2: overexpressed or amplified; Ki-67 proliferation index: any; PR: any); (4) HER2-positive, non-luminal subtype (HER2: overexpressed or amplified; ER: absent; PR: absent); (5) triple-negative subtype (ER: absent; PR: absent; HER2: negative) (Figure 1).

Data on the investigated properties were processed using computer software for statistical analysis, Jandel Sigma Stat 2.0 (SPSS Inc., Chicago, IL, USA) and GraphPad Prism version 5.03 (GraphPad Software, Boston, MA, USA), using options for descriptive statistics, Student’s *t* Distribution, the comparative Fisher’s test and the Pearson correlation test. Differences between groups with a confidence interval of *p* < 0.05 were considered statistically significant.

## 3. Results

**Figure 2:**
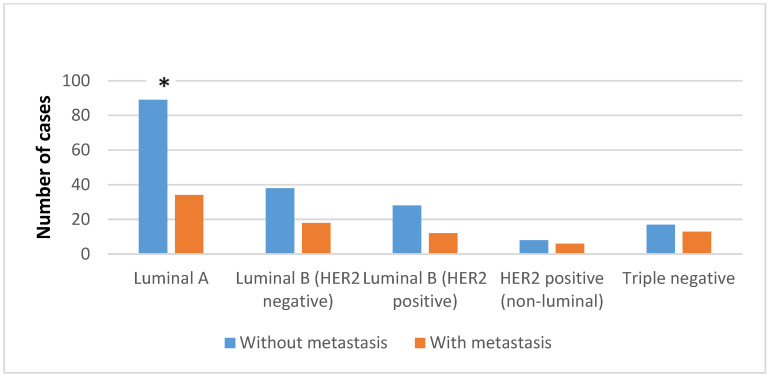
Fisher analysis of data dealing with the positivity of axillary lymph nodes in patients with different molecular subtypes of ILC. * *p* < 0.05.

The molecular subtype luminal A had the highest frequency (46.77%) among 263 cases of breast ILCs that were analyzed in our study. Based on the Student’s *t* test, a statistically significant difference was found between patient age and histological subtype, where patients with solid histological subtype were significantly younger (46 ± 10.9) within the luminal A, luminal B HER2-positive and triple-negative groups compared to other histological subtypes (Table 1, *p* = 0.009). At the same time, the average age of patients with the luminal A subtype was the highest (58.91 ± 11.76) compared to the other molecular subtypes, but without statistically significant differences (Table 1, *p* > 0.01). Also, in relation to the presence of metastases in the axillary lymph nodes, subtype luminal A was singled out with the lowest frequency of metastases (27.64%, *p* = 0.046) compared to all other molecular subtypes, which was statistically significant (Figure 2).

**Figure 3:**
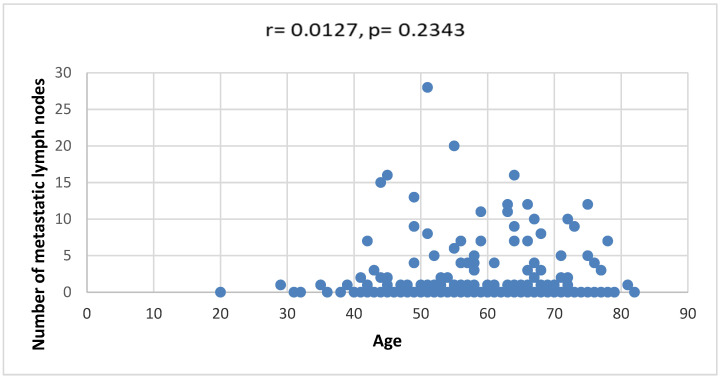
Correlation between the age of patients and the number of metastatic lymph nodes.

The age of female patients showed a weak positive correlation with the number of metastatically changed axillary lymph nodes for all examined ILC subtypes, but without statistical significance (r = 0.012733, *p* = 0.23434) (Figure 3).

**Figure 4:**
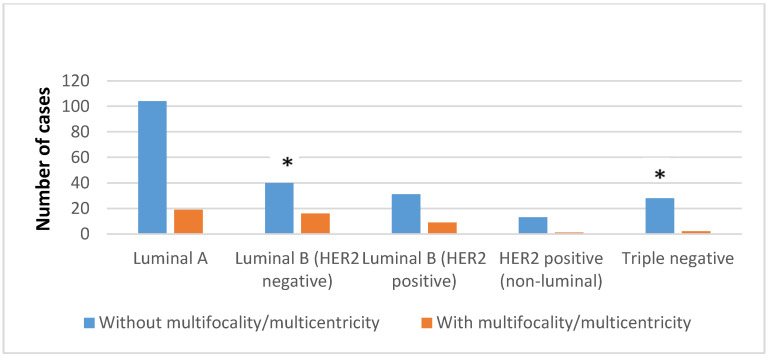
Fisher analysis of data dealing with multifocality/multicentricity in patients with different molecular subtypes of ILC. * *p* < 0.05.

The luminal B HER2-negative molecular subtype had a statistically significantly higher frequency of multifocality/multicentricity (28.57%, *p* = 0.011), while the triple-negative molecular subtype had a statistically significantly lower frequency of multifocality/multicentricity (6.66%, *p* = 0.049) (Figure 4).

HER2-enriched subtype occurs statistically significantly more often in the pT4 stage (28.58%, *p* = 0.024), while the frequency of occurrence of the luminal B HER2-negative subtype is highest in the pT2 stage (53.57%, *p* = 0.03). On the other hand, luminal B HER2-positive subtype occurs least frequently in the pT1 stage (12.5%, *p* = 0.02) and most often in the pT3 (12.5%, *p* = 0.034) or pTx stage (27.5%, *p* = 0.027) (Table 2).

By comparing the histological subtypes of breast ILCs and the molecular subtypes, it was determined that the classic subtype statistically significantly more often occurs as the luminal A subtype (82.93%, *p* = 0.001), and least frequently as the triple-negative molecular subtype (53.33%, *p* = 0.005). On the other hand, the pleomorphic subtype occurred statistically significantly less often as luminal A subtype compared to other subtypes (7.33%, *p* = 0.00027), and significantly more often as the triple-negative subtype (33.33%, *p* = 0.006) or as the luminal B HER2-negative subtype (26.78%, *p* = 0.0069) (Table 3) (Figure 1).

## 4. Discussion

Studies of variations in the molecular subtypes of breast cancer have shown that the frequency of ER-positive breast cancer is the highest, and ranges from 40 to 50% in the luminal A and B subtypes [18], and in other subtypes it can vary significantly and ranges from 10 to 25% in HER2 positive, while it amounts to 13–40% in triple negative [23]. In this study, only breast ILCs were examined in the population that was not covered by regular screening, where ER-positive ILCs were represented by 83.27% and HER2 positive and triple negative accounted for 5.32 and 11.41%, respectively.

Given that the reported frequency of false-negative mammography results is present in 19% of ILC cases [24], in this study, in 16.73% of ILCs, the pT stage could not be determined because the tumor presented as mild architectural distortion of the breast parenchyma, which in some institutions was resolved by an MRI scan after the initial diagnosis of ILC [1].

Reports that MRI is more useful for the diagnosis of multifocal ILC, as well as a higher number of false-positive results due to overestimation of the tumor size [4], may also be related to the results of this study, as luminal B HER2-negative subtypes of ILC were significantly more often multifocal/multicentric (28.57%, *p* = 0.011) and significantly more often in stage pT2 (53.57%, *p* = 0.03), when compared to other molecular subtypes.

Large studies have reported that stage III and IV ILCs greater than 5 cm in diameter were more common in older patients [25,26]. The analysis of molecular subtypes showed that the statistically significant largest ILC diameter was in stage III in luminal B HER2-positive ILCs and in stage IV in HER2 enriched (*p* < 0.05), but at the same time these two subtypes were present in patients of younger age (*p* > 0.05).

The higher metastatic potential of ILC, also reported in older patients [25,26], was not significant for the luminal A subtype of ILC, which had a significantly lower frequency of metastases (*p* = 0.046) and a slightly higher average age of female patients compared to other subtypes (*p* > 0.05).

The distribution of stages depends on race/ethnicity, patient age, ER status and whether breast cancer screening has been performed in the population. There is also a correlation between stage components; for example, only 19% of stage pT1 tumors have axillary lymph node metastases, while as many as 40% of stage pT3 tumors have nodal metastases [7]. The weak positive correlation of patient age with the number of metastatic axillary lymph nodes for all ILC subtypes was without statistical significance (r = 0.012733, *p* = 0.23434).

In most cases, ILCs are classified as low-risk tumors, and due to their generally good prognosis, some authors do not suggest usage of genomic/transcriptional prognostic testing for ILCs and thus recommend the more economical and conventional immunohistochemical classification [15,16]. Gene expression profiling and immunohistochemical studies found that as many as 85% of classical ILCs were classified as luminal A subtype [1], less often as luminal B subtype [19] and rarely as HER2-enriched or triple-negative subtype [20]. In some studies, the higher frequency of luminal B subtypes, HER2-enriched and triple-negative subtypes can be explained by the fact that the analyzed ILCs were diagnosed in the higher stages (pT2, pT3 and pT4) in 58.17% of the cases, as well as by the fact that the progression of low-grade tumors to high-grade tumors primarily occurs in breast tumors that have a luminal phenotype [27,28]. A large number of luminal subtypes (26/41) within pleomorphic ILCs (*p* > 0.01) can be related to some new data that classical and pleomorphic ILC subtypes had similar ER-receptor expression [29]. Also, most of those cases were in the category of luminal B HER2-negative subtype (Table 3). Compared to the luminal A subtype, in the luminal B subtype, the expression of genes associated with ER positivity is low or high for genes associated with proliferation, while it is variable for HER2 expression. This means that luminal A subtypes are likely to benefit from antihormonal therapy, while luminal B subtype tumors are likely to be candidates for additional chemotherapy [1].

Breast cancer positive for hormone receptors can have a range of morphologies and grades [1]. According to literature data, the classical subtype of ILC is predominantly the luminal A molecular subtype [19], which was also confirmed in this study where 82.93% of luminal A subtype were classical ILCs (*p* = 0.001). Many authors have reported that the classical subtype of ILC has a better outcome than other histological subtypes, such as pleomorphic and solid [1,30], which can be related to the results of this study, due to patients with a solid histological subtype being statistically significantly younger compared to other histological subtypes, but only within the luminal A, luminal B HER2-positive and triple-negative molecular subtypes (Table 1, *p* = 0.009).

This study showed that the classic and mixed lobular subtypes account for as much as 79.85% of all histological subtypes of ILC, which is consistent with reports by other authors where they account for the majority (75%) of all histological subtypes of ILC [30].

In general, HER2-positive breast cancers usually have highly pleomorphic nuclei and abundant eosinophilic cytoplasm compared to triple-negative breast cancers [1]; regarding ILC in this study, the pleomorphic subtype was more often HER2 enriched compared to triple negative (*p* > 0.01), but the triple-negative subtype was statistically significantly more often (33.3%, *p* = 0.006) in the pleomorphic subtype category compared to other molecular subtypes.

Approximately 10% to 15% of breast cancers do not express any of these three markers and are called triple-negative breast cancers. They are generally high grade and have a poor prognosis, because there are no benefits from currently available targeted therapeutic modalities [1,13,14]. The nature of triple-negative breast cancers is a high proliferative index [1]. It is also known that the pleomorphic subtype of ILC has a significantly higher mitotic index compared to the classical subtype [1,3]. Some authors have reported that carcinomas with a low percentage of ER-positive cells (and HER2 negativity) often have histological features more similar to high-grade triple-negative carcinomas [31]. These data can be associated with the results of this study, where pleomorphic subtypes of ILC were statistically significantly more often of a triple-negative molecular phenotype compared to other subtypes (33.3%, *p* < 0.01). Also, in this study they may refer to cases of pleomorphic lobular cancers, which were in a significantly smaller percentage (7.3%) of the luminal A molecular phenotype (*p* = 0.0003).

In addition to the majority of cases with positive expression of ER and negative expression of HER2, the luminal B molecular subtype includes a smaller number of breast cancers with co-expression of ER and HER2. The luminal B subtype is usually a higher grade and has a worse prognosis than the luminal A subtype [1]. A Ki-67 proliferative index cut-off of 14% or more correlates with the luminal B subtype versus the less aggressive luminal A subtype. In this sense, this study showed that the pleomorphic subtype was statistically significantly more often the luminal B HER2-negative phenotype than luminal A (26.78% versus 7.33%, *p* < 0.01). Also, a high Ki-67 index of luminal B HER2-negative subtype can be associated with a statistically significantly higher frequency of multifocality/multicentricity in this molecular subtype (Figure 2) (*p* = 0.011). On the other hand, the low Ki-67 proliferative index characteristic of the luminal A subtype [27,32] can be related to the statistically significantly lower metastatic potential of the luminal A subtype in this study (Figure 1) (*p* < 0.05). Some analyses of the Ki-67 proliferative index showed that a lower cut-off value for ILC (4%) is a better marker of prognosis than the above-recommended cut-off value [33]. In general, HER2-positive carcinomas tend to have highly pleomorphic nuclei with abundant eosinophilic cytoplasm compared to triple-negative carcinomas, which is described as apocrine differentiation. The proliferative index is high but, on average, lower compared to triple-negative carcinomas [1], and, in this study, the pleomorphic subtype of ILC had a HER2-enriched molecular phenotype more often than a triple-negative molecular phenotype (35.7% vs. 33.3%), but without a statistically significant difference (*p* > 0.01).

ILCs are mostly low grade and have a good prognosis [15,16,34], which is especially reported for the tubulolobular and alveolar subtypes of ILC, which are considered low-grade tumors [1,35]. This also correlates with molecular subtypes of breast cancer, with classic ILC being predominantly luminal A tumors [19]. In some articles, the pleomorphic subtype of ILC was not analyzed or was probably analyzed as part of the mixed lobular subtype, because the mixed lobular subtype was the HER2-enriched molecular subtype. Also, these articles talk about a trabecular subtype [19,36], which is not described in any classification system. By comparing the histological subtypes of ILC in relation to the molecular classification of ILC, statistical significance was obtained only for two histological patterns, classic and pleomorphic (Table 3). This finding may indicate that cytomorphological characteristics are more important than architectural growth patterns for more detailed profiling of prognostic and predictive parameters. Therefore, reclassification of the histological subtypes of ILC could be considered, primarily according to cytomorphology, such as classic ILC, non-classic ILC and pleomorphic ILC.

## 5. Conclusions

The results of this study significantly singled out the luminal A subtype, and, among them, classic ILC as the subtype with the most favorable expression ratio of the investigated predictive/prognostic immunohistochemical markers. On the other hand, the triple-negative subtype and, among them, pleomorphic ILC significantly had the lowest percentage of multifocal/multicentric presentation. These results indicate that precise and uniform micromorphological classification with adequate immunohistochemical profiling of ILCs can be a good basis for more precise determination of molecular subtypes of ILCs in the future.

## Figures and Tables

**Table 1:** Differences in the average age of patients in relation to molecular subtypes and histological subtypes of ILC.

Average Age	Classic	Alveolar	Solid	Tubulolobular	Pleomorphic	Mixed Lobular	$\bar{x}$
Luminal A-like	58.7 ± 11.5	77	43 ± 4.2	48.7 ± 5.5	62.9 ± 13.4	63.5 ± 10.4	58.9 ± 11.8
Luminal B-like (HER2 negative)	61 ± 10	-	62	-	55.5 ± 13.4	56.2 ± 16	58.5 ± 11.4
Luminal B-like (HER2 positive)	56.6 ± 11.8	54	46 ± 14.1	-	68 ± 14.1	81	57.2 ± 12.5
HER2 positive (non-luminal)	56 ± 5	64	-	-	53 ± 22.5	-	55.5 ± 13.3
Triple negative	60.9 ± 10	-	36	-	54.1 ± 10.5	65.7 ± 6.5	58.3 ± 11.1
$\bar{x}$	58.7 ± 11	65 ± 11.5	46 ± 10.9 *	48.7 ± 5.5	57.1 ± 14.1	62.7 ± 12.6	58.3 ± 11.8

* *p* < 0.01.

**Table 2:** Structure of patients in relation to pT stage and molecular subtype of ILC.

	pT1	pT2	pT3	pT4	pTx	∑
Luminal A-like	35 (28.45%)	55 (44.72%)	4 (3.25%)	10 (8.13%)	19 (15.45%)	123
Luminal B-like (HER2 negative)	15 (26.8%)	30 (53.57%) *	1 (1.78%)	2 (3.57%)	8 (14.28%)	56
Luminal B-like (HER2 positive)	5 (12.5%) *	13 (32.5%)	5 (12.5%) *	6 (15%)	11 (27.5%) *	40
HER2 positive (non-luminal)	2 (14.28%)	4 (28.58%)	2 (14.28%)	4 (28.58%) *	2 (14.28%)	14
Triple negative	9 (30%)	13 (43.33%)	2 (6.67%)	2 (6.67%)	4 (13.33%)	30
∑	66	115	14	24	44	263

* *p* < 0.05.

**Table 3:** Distribution of histological subtypes of ILC in relation to molecular subtypes of ILC.

	Classic	Alveolar	Solid	Tubulolobular	Pleomorphic	Mixed Lobular	∑
Luminal A-like	102 (82.93%) *	1 (0.81%)	2 (1.63%)	3 (2.42%)	9 (7.33%) *	6 (4.88%)	123
Luminal B-like (HER2 negative)	35 (62.5%)	0 (0%)	1 (1.79%)	0 (0%)	15 (26.78%)	5 (8.93%)	56
Luminal B-like (HER2 positive)	34 (85%)	1 (2.5%)	2 (5%)	0 (0%)	2 (5%)	1 (2.5%)	40
HER2 positive (non-luminal)	8 (57.14%)	1 (7.15%)	0 (0%)	0 (0%)	5 (35.71%)	0 (0%)	14
Triple negative	16 (53.33%) *	0 (0%)	1 (3.34%)	0 (0%)	10 (33.33%) *	3 (10%)	30
∑	195	3	6	3	41	15	263

* *p* < 0.01.

## Data Availability

Data are contained within the article.
